# Isolated neonatal bilateral vocal cord paralysis revealing a unilateral medullary defect: a case report

**DOI:** 10.1186/s12887-018-1329-y

**Published:** 2018-11-09

**Authors:** Camille Brotelande, Nicolas Leboucq, Mohamed Akkari, Thomas Roujeau, Massimo Di Maio, Christophe Milési, Michel Mondain, Charles Raybaud, Gilles Cambonie

**Affiliations:** 1 Department of Neonatology and Paediatric Intensive Care Unit, Arnaud de Villeneuve Hospital, Montpellier University Hospital, 371 Avenue du Doyen Gaston Giraud, 34295 Montpellier, Cedex 5 France; 2 Department of Paediatric Radiology, Arnaud de Villeneuve Hospital, Montpellier University Hospital, Montpellier, France; 3 Department of Otorhinolaryngology, Gui-de-Chauliac Hospital, Montpellier University Hospital, Montpellier, France; 4 Department of Neurosurgery, Gui-de-Chauliac Hospital, Montpellier University Hospital, Montpellier, France; 5 Neonatal Intensive Care Unit, Carémeau Hospital, Nîmes University Hospital, Nîmes, France; 6 Division of Neuroradiology, Hospital for Sick Children, University of Toronto, 555 University Avenue, Toronto, ON M5G1X8 Canada

**Keywords:** Bilateral vocal cord paralysis, Brainstem, Newborn

## Abstract

**Background:**

Congenital bilateral vocal cord paralysis is a rare occurrence. Approximately half the cases are associated with a major comorbidity, usually neurological, neuromuscular or malformative.

**Case presentation:**

In a male newborn, respiratory distress syndrome and stridor were observed immediately following birth. The cause was bilateral vocal cord paralysis in the adducted position. Neuroradiological investigation revealed a unilateral discontinuity between the upper pons and the right medulla oblongata. Hypoplasia of the right posterior hemiarches of C1-C2 and the right exo-occipital bone was observed, as was a small clivus. MR angiography showed the absence of the distal right vertebral artery, with hypoplasia and parietal irregularities of the proximal segments. Respiratory autonomy was not obtained despite endoscopic laser cordotomy, corticosteroid therapy and nasal continuous positive airway pressure. The infant died at the age of 4 weeks after treatment was limited to comfort care.

**Conclusions:**

A medullary lesion is an exceptional cause of congenital bilateral vocal cord paralysis. The strictly unilateral neurological and vascular defect and the absence of associated intracranial or extracranial malformation make this clinical case unique and suggest a disruptive mechanism. This case also highlights the help provided by advanced neuroimaging techniques, i.e. fibre tracking using diffusion tensor imaging, in the decision-making process.

## Background

Bilateral vocal cord paralysis (BVCP) is very rare in the neonate, with the incidence estimated at 0.75 cases per million births per year [1]. The series focused on congenital BVCP have highlighted two fairly well-balanced aetiological groups: one idiopathic and the other associated with major comorbidities [2–4]. The latter group includes mainly neonates with perinatal encephalopathy, neuromuscular diseases, chromosomal or genetic anomalies and major malformations [3, 5]. The malformations are generally intracranial in this context. Chiari 1 is the “classic” condition, but choroid plexus cysts, ventriculomegaly, hydrocephalus, myelomeningocele, and brainstem dysgenesis have also been described [3, 6]. We report an exceptional cause of isolated congenital BVCP, a unilateral medullary defect, and discuss its pathogenesis and the management strategy adopted in this specific case.

## Case presentation

This boy was born at 37^+ 2^ weeks of gestation from a 30-year-old mother. He was the first child of non-consanguineous and healthy Caucasian parents. The patient’s mother received prenatal care during pregnancy. At 12 weeks, Doppler ultrasound (US) examination revealed increased resistance in the uterine arteries, and salicylic acid (100 mg daily) was prescribed. From the 23rd week, several Doppler US scans showed intrauterine growth retardation with persistent notching in the right uterine artery and increased resistance in the left. No scan revealed a malformation. Labour was spontaneous and the boy was born by vaginal delivery using Thierry’s spatula because of abnormal foetal heart rate. Apgar scores were 4, 7 and 8 at 1, 5 and 10 min, respectively; arterial cord blood pH was 7.22 and cord lactate was 5.1 mmol/L. Birth weight was 2045 g (0.6th centile, according to customized French curves), length was 51 cm (91th centile), and head circumference was 32 cm (10th centile). The placenta was hypotrophic (280 g), with peripheral insertion of the cord and signs of maternal vascular hypoperfusion, but no lesion of decidual arteriopathy at pathological examination.

Respiratory distress, including suprasternal tugging and stridor, was observed immediately following birth. The neonate was bagged with air for a few minutes and then supported with nasal continuous positive airway pressure (CPAP). Prolonged apnoea associated with bradycardia required caffeine from the first postnatal day.

The first series of exams showed normal brain US and normal serum electrolytes with calcium. Flexible fibreoptic laryngoscopy (FFL) performed on day 2 revealed BVCP in the adducted position, causing severe airway obstruction and prompting transfer to a medical and surgical neonatal intensive care unit on day 3.

On admission, clinical examination revealed a wide anterior fontanel, enlarged coronal sutures, normal temperature, and persistent respiratory distress despite CPAP. Facial and ocular motricity were apparently normal, and the sucking reflex was present. The newborn was intubated a few hours later for worsening respiratory distress and severe apnoea. Rigid laryngotracheal endoscopy performed on day 4 under general anaesthesia showed neither malformation nor other obstruction below the glottis. Chest X-ray and abdominal and cardiac US were normal.

**Fig. 1:**
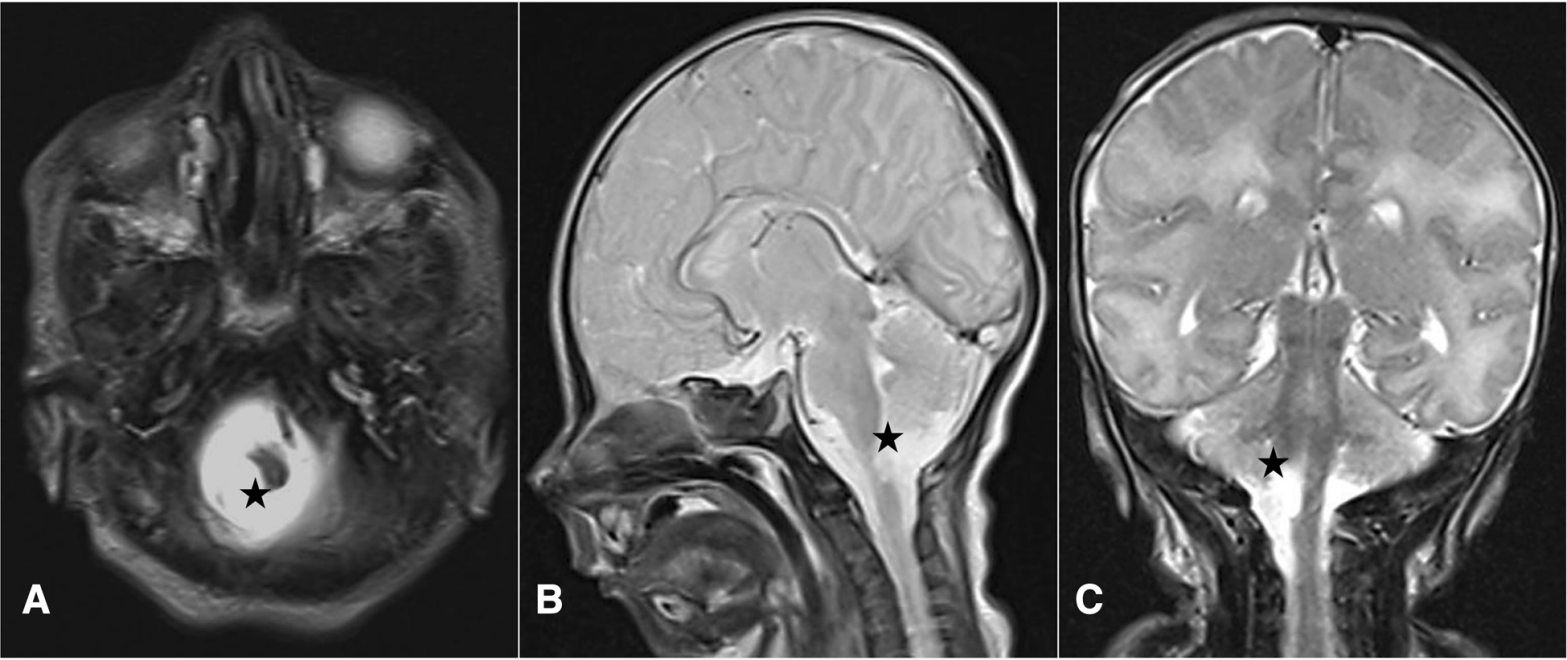
Cerebral MRI. Axial (**a**), Sagittal (**b**) and Coronal (**c**) T2-weighted sequence showing deformation of the cervicomedullary junction with a focal defect within the right medulla oblongata (black stars)

**Fig. 2:**
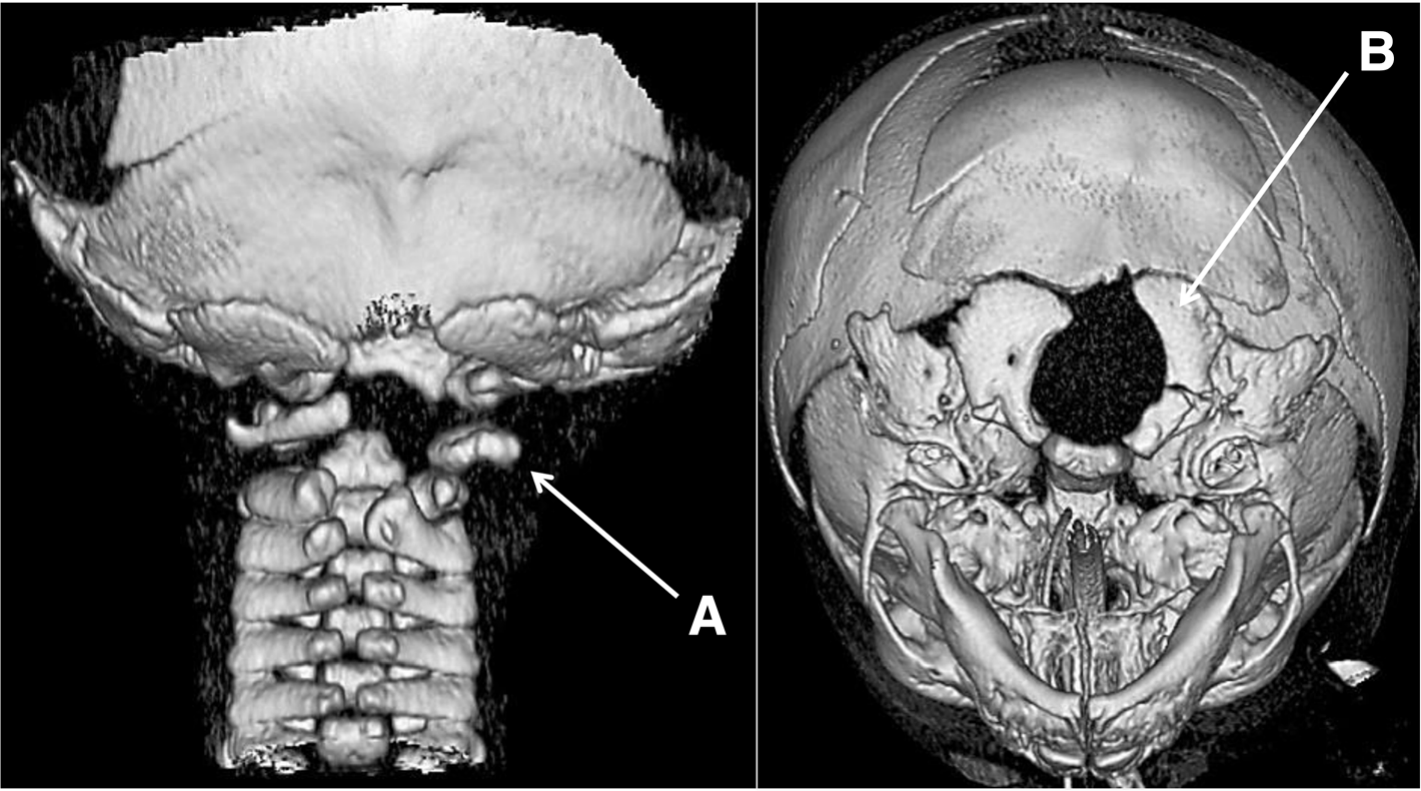
3D bone CT reconstruction showing hypoplasia of the posterior hemiarches of C1-C2 (**a**) and the right exo-occipital bone (**b**) and a small clivus

**Fig. 3:**
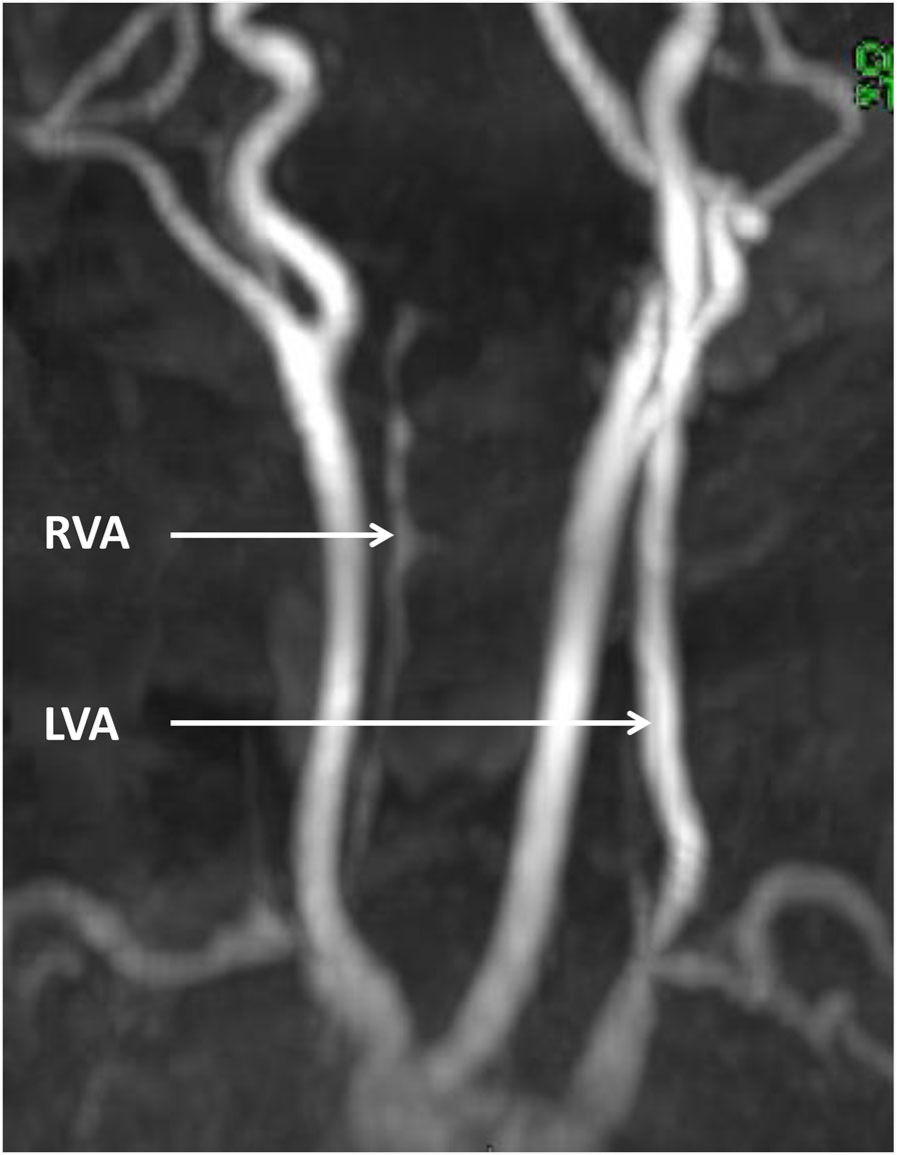
MR angiography showing hypoplasia and parietal irregularities of the V1 and V2 segments (white arrow) and agenesis of the V3 and V4 segments of the right vertebral artery (RVA). LVA: left vertebral artery

**Fig. 4:**
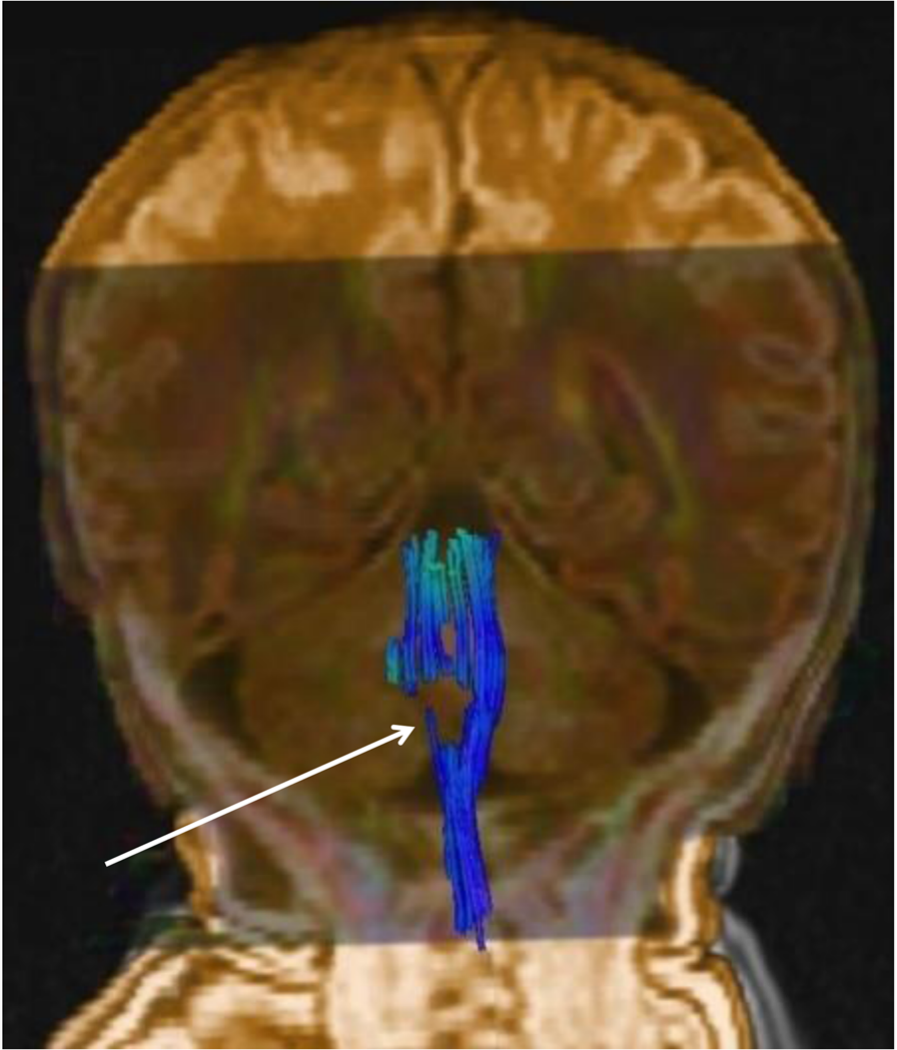
Fibre tracking using diffusion tensor imaging showing the section of the right lateral and median sensorimotor fascicles (white arrow)

Neuroradiological investigations were performed between day 4 and day 15. The first brain MRI showed a deformation of the bulbo-medullary junction with an arched aspect in the anteroposterior direction slightly deflected to the left. Enlarged subarachnoid spaces and foramen magnum surrounded this junction. Cortical structures were normal, myelination was age-appropriate, and the pituitary axis and corpus callosum were normal (Fig. 1). Additional investigations were conducted, with MRI and CT focused on the cervico-occipital hinge. MRI showed a unilateral cavitated lesion, with discontinuity between the upper pons and the medulla oblongata. CT revealed vertebral anomalies, with hypoplasia of the right posterior hemiarches of C1-C2, hypoplasia of the right exo-occipital bone and a small clivus (Fig. 2). On day 15, MR angiography showed the absence of the distal right vertebral artery (Fig. 3). MRI with fibre tracking, using diffusion tensor imaging, confirmed hemisection of the right lateral and median sensorimotor fascicles (Fig. 4). Brainstem auditory evoked potentials and fundus examination were normal.

Patient management included a left unilateral endoscopic laser cordotomy on day 7 in order to proceed to extubation on day 8. The procedure was unsuccessful despite corticosteroid therapy and CPAP, and the infant was reintubated after 3 h due to the reappearance of stridor and major signs of respiratory distress. Mechanical ventilation was maintained until the end of the stay. The severity of this situation prompted a multidisciplinary ethics consultation and the decision was made to limit care to the infant’s comfort. The parents were informed and agreed with this decision, given the very poor respiratory and neurosensory prognosis. The infant died at the age of 4 weeks. An autopsy was not performed. Array comparative genomic hybridization revealed no abnormality.

## Discussion

BVCP is generally diagnosed in neonates by the end of the first postnatal month [3]. Stridor is always a presenting feature, associated with respiratory difficulties of variable severity in 80–90% of the cases [7]. Direct visualization of the pharynx and larynx using FFL should be systematic to investigate stridor and enable dynamic study of vocal cord motility. This examination needs to be completed with rigid laryngotracheal endoscopy under general anaesthesia, for subglottic and tracheal assessment [8]. Other methods for exploring the laryngeal region have been proposed, but they are not routinely used in most centres. Laryngeal US provides dynamic morphometric parameters, and it may offer a less invasive means of evaluating vocal fold motility in critically ill neonates, which is particularly interesting for the follow-up of paralysis [9, 10]. Laryngeal electromyography can also help to better characterize laryngeal motility disorders and anticipate the prognosis of bilateral laryngeal palsies. Interpretation, however, is not fully standardized in neonates and children [11].

Unilateral or BVCP may occur in neonates following thoracic surgery, including cardiovascular surgery - mainly patent ductus arteriosus ligation [12] - and surgery for oesophageal atresia and/or tracheo-oesophageal fistula [13]. Outside of this context, additional examinations are needed to determine the underlying cause [14]. MRI of the brain, neck, mediastinum and chest provide detailed anatomical study of the brainstem, larynx, and the vagus and recurrent laryngeal nerves [15].

The central neurological disorders or anatomic abnormalities potentially associated with BVCP, including Chiari malformation alone or associated with myelomeningocele and hydrocephalus, can be detected on prenatal US examination. Williams syndrome, Moebius syndrome, congenital myasthenic syndrome, 22q deletion syndrome, and Down syndrome have occasionally been associated with BVCP in the neonatal period. Small series of familial congenital BVCP with variable inheritance patterns and chromosomal alterations have also been described [16].

In our case, MRI revealed a unilateral medullary defect, which has been referenced in the medical literature under the terms brainstem disconnection or brainstem disruption. Brainstem disconnection is a very rare congenital anomaly of the infratentorial region, defined by an absent brainstem segment. This regional discontinuity may be observed at the pontomesencephalic level but is more common at the pontomedullary level [17]. Most reports have described extensive defects, with only a thin cord of tissue between the rostral and caudal brainstem portions [18]. An asymmetrical defect or, as in our case, a unilateral defect is quite exceptional, having been reported only once within the left medulla oblongata and described by the authors as a brainstem disruption [19].

Prenatal diagnosis with foetal MRI has exceptionally been performed in cases of brainstem disconnection [20]. Polyhydramnios has been reported, probably in relation with impaired prenatal swallowing. In our patient, the sucking reflex was present, but it was difficult to determine whether deglutition was normal. In addition, the polyhydramnios may have been neutralized by the impaired uteroplacental perfusion. Cerebellar hypoplasia, sometimes detected with foetal US, has been observed in all patients with a severe defect. Other brain abnormalities have been inconsistently mentioned: absence of cerebellar peduncles, hypoplastic or absent optic nerves, absence of internal auditory canals, dilated cisterns or cerebral ventricles, periventricular nodular heterotopia, and hamartoma [20]. Extracerebral abnormalities, sometimes multiple in a single patient [21] and notably involving the axial skeleton, heart, and digestive and genitourinary tracts, have been noted in both pontomesencephalic and pontomedullary disconnections [17].

Arguments in favour of a genetic cause have mainly been raised for bilateral lesions and extensive forms associated with malformations, such as the neuronal migration disorders [22]. The absence of gliotic lesions on histological examination has also been interpreted in favour of this aetiology [23]. From this perspective, the brainstem dysgenesis results from an abnormal expression of segmentation genes or their upstream modulators at the midbrain-hindbrain boundary [17, 18]. Animal models, however, do not unequivocally reproduce segmental dysgenesis [24], and no mutation of candidate genes, such as EN2, or causative variations on whole exome sequencing have thus far been documented in humans [17, 22, 23].

Focal lesions, whether associated or not with abnormalities of the vertebrobasilar arterial system, have led to the alternative hypothesis of an acquired, i.e. disruptive, mechanism [19]. The previously described vascular abnormalities in brainstem disconnection were mainly the absence or the extreme hypoplasia of the vertebral or basilar arteries [20]. In our case, MR angiography demonstrated hypoplasia and parietal irregularities of the proximal segments and absence of the distal segments of the right vertebral artery. The unilateral, well-defined, elliptical anteroposterior lesion of the cervico-medullary junction in our patient may be consistent with intrauterine ischaemia, but the precise timing of the insult is difficult to establish. During the early embryonic period, the posterior circulation is exclusively supplied by the internal carotid arteries through a series of anastomoses that will regress with the development of the vertebrobasilar arterial system [25]. The trigeminal, otic and hypoglossal arteries intervene in the formation of the basilar artery. The cervical portion of the vertebral artery, i.e. the V1 and V2 segments, is formed by anastomoses from the first six cervical intersegmental arteries, while the proatlantal intersegmental artery contributes to the formation of the V3 and V4 segments. In our patient, the lack of visualization of the V3 and V4 segments of the right vertebral artery on MR angiography may be explained by early occlusion or agenesis of the proatlantal artery. However, the origins of these vascular abnormalities may also be dysplastic or inflammatory, in parallel with the vascular involvement observed as early as 12 weeks of gestation at the level of placenta. Moreover, the resulting ischaemia may have caused the focal defect at the medullary level, the homolateral hypoplasia of the exo-occipital bone, and the lateral mass of C1. Indeed, these last two elements probably have a common embryological origin, from the fourth occipital and the first spinal sclerotomes, and they are also perfused by the proatlantal artery [26]. The hypoplasia of the right transverse process of C2 might be related to the hypoplasia of segments V1 and V2, its blood supply being provided by the first intersegmental cervical artery [27]. Other causes of disruptions, such as alcohol or cocaine exposure during pregnancy, have also been described in brainstem disconnection [28, 29]. Nevertheless, the controversy concerning a malformative or vascular origin of this condition has not yet been resolved. The involvement of genes critical for both the formation of the brainstem and its vascularization should also not be excluded [22].

In our patient, the initial decision for endoscopic surgical treatment was taken before MRI with fibre tracking demonstrated the unilateral medullary defect. However, neither this treatment nor CPAP, which can potentiate the results of an endoscopic procedure [4], prevented rapid reintubation. Several elements were taken into account in the multidisciplinary decision to limit curative care. The prognosis of brainstem disconnection is generally very poor, with respiratory failure occurring soon after support is removed. Death generally occurs in the first 2 months following birth, but cases of prolonged survival under chronic respiratory assistance have occasionally been described [22, 30]. Indeed, functional respiratory outcome is very poor in the event of congenital BVCP associated with major underlying comorbidity, with resolution of the vocal cord immobility in only 14% of patients and decannulation after 2 to 3 years in 25% [3, 31]. More recently, survival without mechanical ventilation has also been reported; however, gastrostomy was systematically needed, as well as tracheostomy at times [17, 19]. No or minimal achievement of developmental milestones has been noted in children with large defects [17]. In our case, characterized by a rather well-circumscribed, unilateral defect, tractography was particularly useful for reconstructing the course of the fibre tracts within the brainstem [32]. This microstructural analysis highlighted a section with several bundles, including the corticospinal, spinothalamic and spinocerebellar tracts, and the medial lemniscus. In addition, the topography of the lesion suggested the involvement of parts of the spinal tract of the right trigeminal nerve, the right nucleus tractus solitarii, the right cuneiform nucleus and the medullary reticular formation. In our patient, the BVCP also expressed the involvement of the ambiguus nuclei, which intervene in the motor innervation of the laryngeal muscles and adjoin the median raphe at the lower part of the medulla. Thus, relatively small lesions of the brainstem can have dramatic consequences, as nicely expressed by Boltshauser et al.: “small lesions-big problems” [19]. These authors reported on two children, aged 19 months and 32 months, with unilateral or asymmetrical defects. The examinations confirmed the multiple and major consequences of these lesions, in particular swallowing impairment, cranial nerve deficits, hemiparesis, and motor, speech and cognitive delays [19].

## Conclusion

An isolated BVCP in a neonate can be caused by a medullary defect, a very rare congenital anomaly. This case report contributes to the phenotypic description of this poorly understood entity, here characterized by a strictly unilateral neurological and vascular defect and the absence of associated malformation. It also highlights the complexity of the decision-making process and the help provided by advanced neuroimaging techniques to analyse the hindbrain region.

## Abbreviations

BVCP: Bilateral vocal cord paralysis
C1: 1st cervical vertebra
C2: 2nd cervical vertebra
CPAP: Continuous positive airway pressure
CT: Computed tomography
FFL: Flexible fibreoptic laryngoscopy
MRI: Magnetic resonance imaging
US: Ultrasound
